# Motor neuron apoptosis and neuromuscular junction perturbation are prominent features in a *Drosophila *model of Fus-mediated ALS

**DOI:** 10.1186/1750-1326-7-10

**Published:** 2012-03-24

**Authors:** Ruohan Xia, Yajuan Liu, Liuqing Yang, Jozsef Gal, Haining Zhu, Jianhang Jia

**Affiliations:** 1 Markey Cancer Center, University of Kentucky, KY 40536 Lexington, USA; 2 Department of Molecular and Cellular Biochemistry, College of Medicine, University of Kentucky, KY 40536 Lexington, USA

**Keywords:** ALS, Fus, Caz, Locomotion, Neurodegeneration, *Drosophila*

## Abstract

**Backgound:**

Amyotrophic lateral sclerosis (ALS) is progressive neurodegenerative disease characterized by the loss of motor function. Several ALS genes have been identified as their mutations can lead to familial ALS, including the recently reported RNA-binding protein fused in sarcoma (Fus). However, it is not clear how mutations of Fus lead to motor neuron degeneration in ALS. In this study, we present a *Drosophila *model to examine the toxicity of Fus, its *Drosophila *orthologue Cabeza (Caz), and the ALS-related Fus mutants.

**Results:**

Our results show that the expression of wild-type Fus/Caz or FusR521G induced progressive toxicity in multiple tissues of the transgenic flies in a dose- and age-dependent manner. The expression of Fus, Caz, or FusR521G in motor neurons significantly impaired the locomotive ability of fly larvae and adults. The presynaptic structures in neuromuscular junctions were disrupted and motor neurons in the ventral nerve cord (VNC) were disorganized and underwent apoptosis. Surprisingly, the interruption of Fus nuclear localization by either deleting its nuclear localization sequence (NLS) or adding a nuclear export signal (NES) blocked Fus toxicity. Moreover, we discovered that the loss of *caz *in *Drosophila *led to severe growth defects in the eyes and VNCs, caused locomotive disability and NMJ disruption, but did not induce apoptotic cell death.

**Conclusions:**

These data demonstrate that the overexpression of Fus/Caz causes *in vivo *toxicity by disrupting neuromuscular junctions (NMJs) and inducing apoptosis in motor neurons. In addition, the nuclear localization of Fus is essential for Fus to induce toxicity. Our findings also suggest that Fus overexpression and gene deletion can cause similar degenerative phenotypes but the underlying mechanisms are likely different.

## Background

Amyotrophic lateral sclerosis (ALS, also known as Lou Gehrig's disease) is a motor neuron disease that causes a disabling condition whereby the degeneration of motor neurons causes progressive muscle weakness and leads to death usually within five years of disease onset [1]. Most cases of ALS occur sporadically, however a small percentage of cases are inherited. Several ALS genes have been identified, and mutations within them can lead to familial ALS [2], including Cu/Zn superoxide dismutase (SOD1) [3], TDP-43 [4] and Fused in sarcoma (Fus) [5-8]. In addition to familial ALS, Fus mutations are also implicated in sporadic ALS cases [9-11]. Both Fus and TDP-43 are DNA- and RNA-binding proteins. While TDP-43 has been intensively studied, the role of Fus in the ALS etiology is largely unknown.

Fus is a ubiquitous multi-domain RNA-binding protein [12] involved in many processes of RNA metabolism including transcriptional regulation, mRNA splicing and mRNA shuttling between the nucleus and the cytoplasm [13,14]. In neurons, Fus is also implicated in the transportation of mRNA for local translation in dendrites [15,16]. Fus mainly localizes in the nucleus, but has also been shown to present at lower levels in the cytoplasm of most cell types, including neurons and glial cells [17,18]. The ALS-related mutations are clustered in the carboxyl-terminus of Fus and exhibit an abnormal nucleo-cytoplasmic redistribution and cytosolic inclusions in the motor neurons in familial ALS patients [7,8]. Studies at the cellular level have shown that the nuclear localization sequence (NLS) located in the C-terminus of Fus is necessary and sufficient for nuclear targeting of Fus [19]. ALS mutations within the NLS significantly impair the nuclear targeting function of the sequence, leading to the cytoplasmic accumulation of mutant Fus. Mutations in the NLS have also been shown to promote Fus co-localization with stress granules in the cytosol [19-21]. However, it is still unclear how the mutations cause the toxicity. Moreover, wild-type Fus aggregation has also been reported in several ALS cases. Thus, it is important to determine whether the cytoplasmic or nuclear fraction of Fus is critical to cause the toxicity in ALS. The tentative hypothesis in the field is that cytoplasmic mislocalization and inclusions are important during the disease process. However, this remains to be thoroughly tested.

Appropriately constructed animal models are valuable for defining the link between gene mutations and the ALS disease. Well-established genetics, small genome size, minimal genetic redundancy, and ease of handling have made *Drosophila *an ideal model system to study conserved mechanisms that are involved in human diseases. Studies using *Drosophila *as a model have shown that mutating TDP-43 causes locomotive defects [22], whereas overexpressing TDP-43 causes neurotoxicity [23], most likely through genetic interactions with TER94, which is an ATPase associated with multiple cellular activities [24]. These findings suggest that *Drosophila *is a powerful animal model for ALS. Studies of transgenic *Drosophila *lines with the overexpression of Fus have been reported in the past [25-27], but the molecular mechanisms underlying Fus/Caz function in *Drosophila *remain elusive. The *Drosophila *homologue of Fus, Cabeza (Caz), is enriched in the brain and central nervous system [28,29]. However, the functional link between *Drosophila *Caz and human Fus has not yet been established.

In this study, we generated and characterized transgenic flies expressing wild-type Fus, several ALS-related Fus mutants, and wild-type Caz using different tissue-specific Gal4 lines. We found that toxicity was dependent on the expression level of Fus/Caz in all tissues examined. Flies overexpressing Fus or Caz by the motor neuron-specific Gal4 showed significantly impaired larval crawling and adult fly movement. Defects in the neuromuscular junctions (NMJs) were evident in these flies. In addition, motor neurons in the ventral nerve cord (VNC) of these flies were disorganized and showed elevated apoptosis. More interestingly, C-terminal NLS is essential for Fus toxicity, since both the deletion of the last 32 amino acids in Fus (FusΔ32) and the addition of an NES to wild type full-length Fus (FusNES) dramatically reduced toxicity *in vivo*. Finally, we analyzed the *caz *mutant flies and found that the loss of Caz induced phenotypes in *Drosophila *eyes, VNC neurons, and NMJs. The lack of Caz in motor neurons caused similar NMJ defects and locomotive impairment as the Fus/Caz overexpressing flies, but did not cause apoptosis in motor neuron cell bodies. Thus, the mechanisms underlying the toxicity caused by Caz deletion and Fus/Caz overexpression are likely different. The genetic model we generated shows that Fus/Caz is required for neuronal function *in vivo *and that elevated levels of Fus/Caz in the nucleus can induce neuronal toxicity. The findings of this study provide insights into the molecular mechanisms of Fus/Caz in motor neuron degeneration and ALS.

## Results

### The expression of Fus/Caz results in wing defects and decreased viability in *Drosophila*

Animal models for neurodegenerative diseases often involve overexpression of disease relevant proteins in yeast, *Drosophila, C. elegans*, and mouse. We generated transgenic flies with the PhiC31 integration system to integrate human Fus or *Drosophila *homolog Caz cDNA at the 75B1 attP locus in the *Drosophila *genome to ensure that the proteins were expressed at the same level without positional effects [30,31]. Using this precise approach, we investigated the pathogenic properties of human Fus and fly Caz in *Drosophila*. Individual Fus variants were overexpressed in a tissue-specific fashion using (i) a wing-specific *MS1096*-Gal4, (ii) a ubiquitous *act5C-*Gal4 in all fly tissues, (iii) an embryonic nervous system specific *Elav*-Gal4, and (iv) a larval and adult motor neuron-specific *D42*-Gal4.

**Figure 1:**
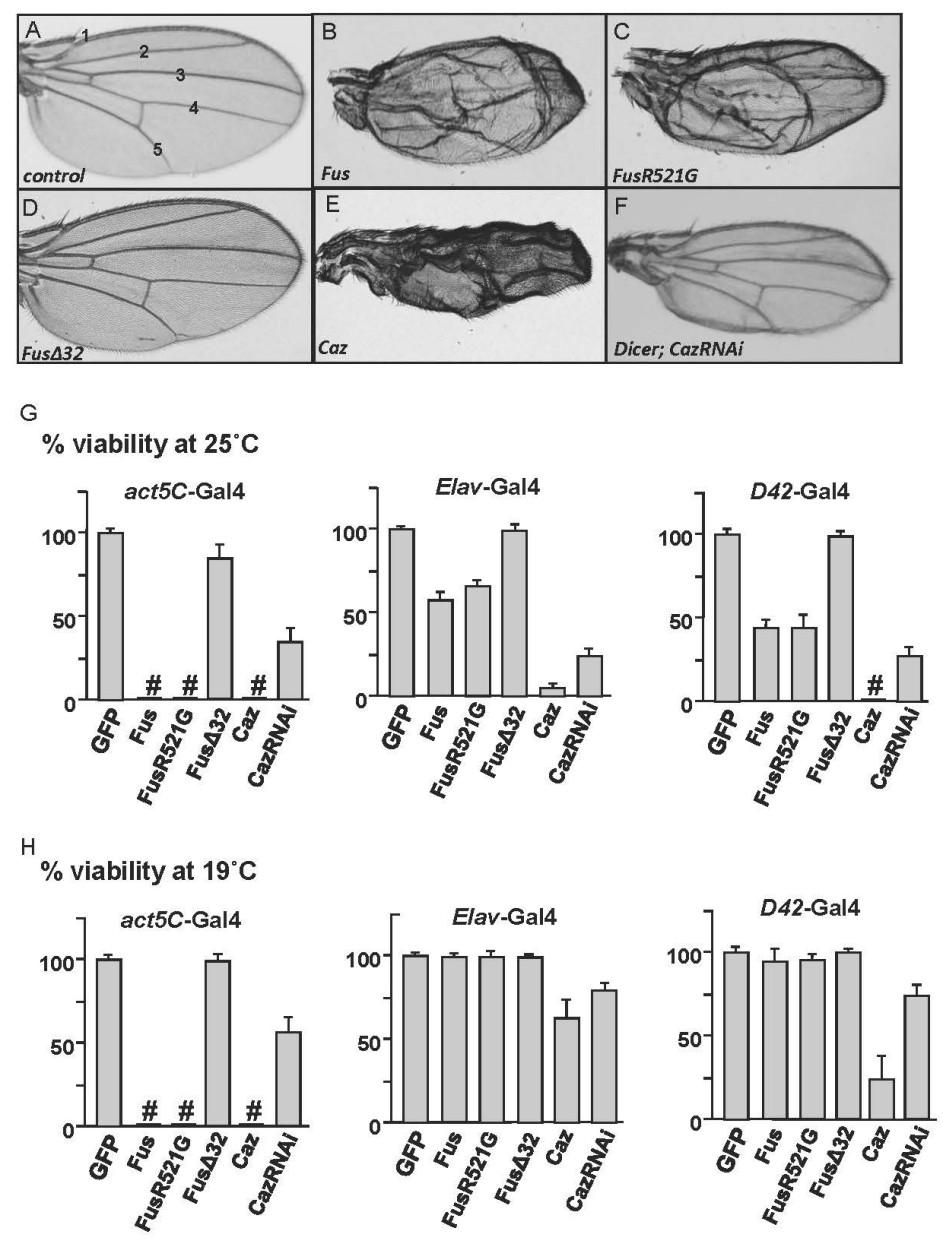
**Overexpression of Fus/Caz produces phenotypes and induces growth defects in *Drosophila***. (A) A wild-type adult wing showing intervein 1-5. (B-F) Adult wings expressing Fus, FusR521G, FusΔ32, Caz, or CazRNAi with *UAS-Dicer *by the wing-specific *MS1096*-Gal4. (G) Bar graphs showing adult fly viability assayed at 25°C. (H) Bar graphs showing adult fly viability assayed at 19°C. Overexpressing the indicated constructs by different Gal4 lines induces growth defects or lethality at larval or pupal stages. The # indicates lethality at either the larval or pupal stages.

We first found that overexpressing Myc-tagged Fus (Myc-Fus) or FusR521G mutant (Myc-FusR521G) using *MS1096*-Gal4 interrupted wing growth (Figure 1B-C). The ubiquitous overexpression of Fus severely reduced adult viability at 25°C and 19°C (Figure 1G-H). Overexpressing Fus in neurons using *Elav*-Gal4 and *D42*-Gal4 also significantly reduced adult viability at 25°C (Figure 1G), but the adult viability improved at 19°C when the Fus overexpression level was lower (Figure 1H). The overexpression of Myc-FusR521C produced similar phenotypes to those caused by overexpressing Myc-FusR521G (data not shown). Surprisingly, we found that expressing Myc-tagged Fus lacking the last 32 amino acids of the NLS (Myc-FusΔ32) gave rise to a wild-type wing (Figure 1D) and nearly 100% adult viability (Figure 1G-H). These data give the first indication that the C-terminal NLS is required for Fus toxicity *in vivo*.

Caz is the *Drosophila *homologue of Fus and has been reported to be enriched in the brain and central nervous system [28,29]. Caz shares a similar domain structure with Fus (Additional file 1: Figure S1) and exhibits 51% identity to Fus in the C-terminal region containing the RNA recognition motif and RGG domains. However, its *in vivo *function and its possible role in neurodegeneration are unclear. We examined the potential role of Caz in neurodegeneration using overexpression, RNAi knockdown, and knockout approaches in this study. The overexpression of HA-tagged *Drosophila *Caz (HA-Caz) caused similar phenotypes in the wing (Figure 1E) and even more dramatic decrease in adult viability (Figure 1G-H). The native Caz protein may adopt a more favorable condition in the flies and therefore induce more severe toxicity compared to overexpressed human Fus. These findings suggest that the overexpression of Fus or Caz had similar growth defects in flies. Interestingly, the knockdown of Caz by RNAi affected wing growth (Figure 1F) and reduced adult viability (Figure 1G-H), suggesting that endogenous Caz is involved in *Drosophila *growth control. This provides initial evidence that Caz deletion can produce toxicity as well, which was thoroughly examined in *caz *mutant flies later in this study.

### Over-expression of Fus/Caz in *Drosophila *eye induces retinal degeneration

**Figure 2:**
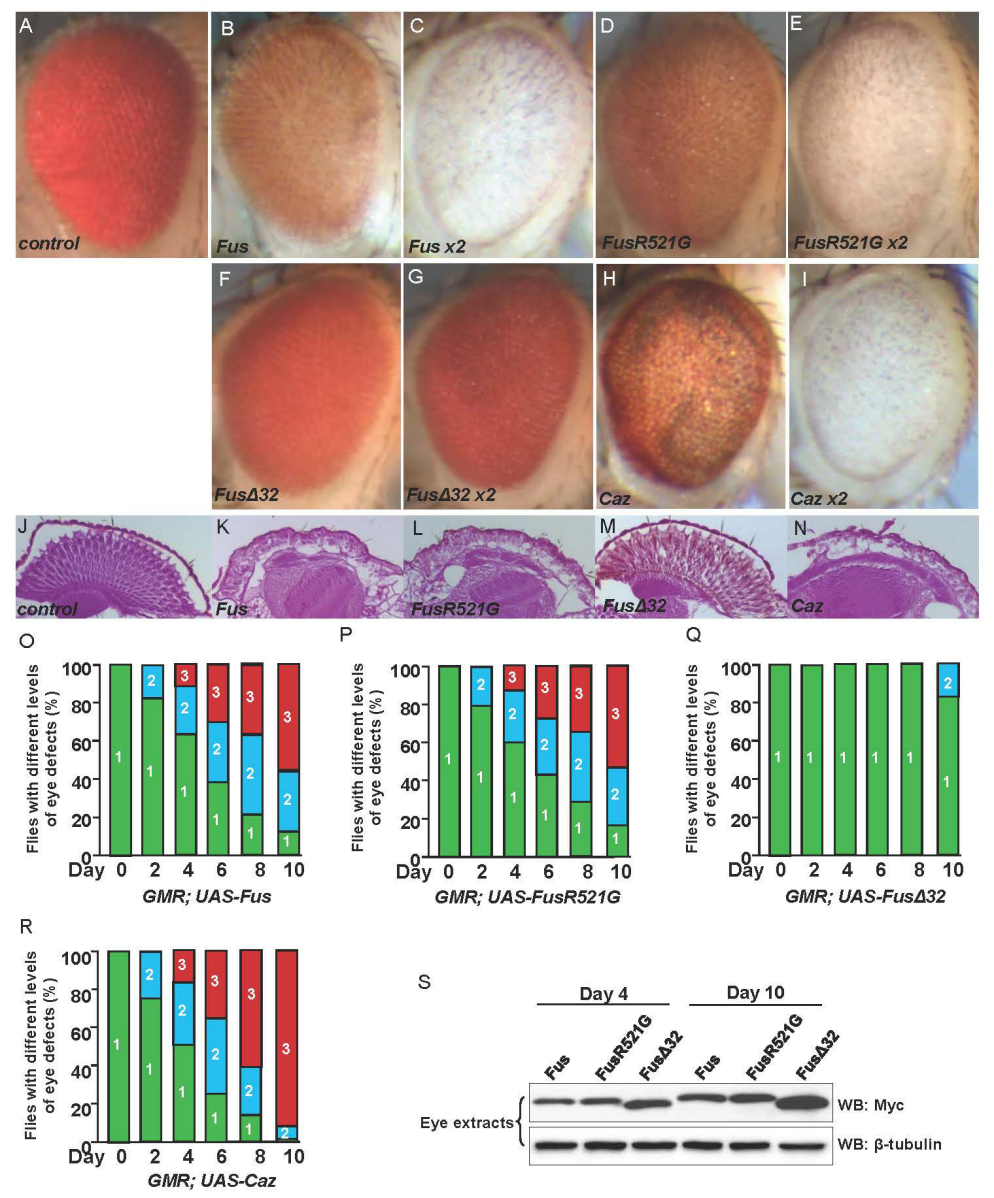
**Expression of Fus/Caz in fly eyes leads to progressive eye defects in a dose- and age-dependent manner**. (A) A wild-type eye showing the typical ommatidia structure. (B-I) Morphology of eyes expressing one or two copies of Fus, FusR521G, FusΔ32, or Caz by *GMR*-Gal4 at day 4 after adult hatching. Fus, FusR521G, and Caz expressing eyes exhibit ommatidia loss and necrotic lesions. (J) Retinal section of a wild-type fly eye stained with Eosin shows intact ommatidial and rhabdomere structure. (K-N) Retinal sections of eyes expressing two copies of Fus, FusR521G, FusΔ32, or Caz by *GMR*-Gal4 at 4 DAE were stained with Eosin. Rhabdomere structures were disintegrated and ommatidia were disorganized in eyes expressing Fus, FusR521G, or Caz, but not in eyes expressing FusΔ32. (O-R) Quantification of the eye defects when expressing the indicated constructs during aging. Eye defects were classified in three levels: 1, < 30% ommatidia loss; 2, 30-70% ommatidia loss, and 3, > 70% ommatidia loss. The percentages of flies with each level of eye defects are shown in different colors (1, green; 2, blue; 3, red). (S) Western blots were performed with eye extracts from flies in Figure 2O-R at the indicated time points to monitor the levels of protein expression. Each individual protein was expressed at the attP site of the 75B1 locus by *GMR*-Gal4 to ensure that the proteins were expressed at a comparable level.

The *Drosophila *compound eye, which consists of approximately 750 single eye units called ommatidia, has been used as a model for studying human neurodegenerative disorders [32]. We used a *GMR*-Gal4 driver to express Myc-Fus, FusR521G, FusΔ32, and HA-Caz in *Drosophila *eyes. We found that the expression of Fus (Figure 2B-C) or Caz (Figure 2H-I) caused severe eye degeneration. The expression of FusR521G induced a phenotype similar to that caused by Fus (Figure 2D-E). We also examined eye structures of the frontal sections using Eosin Phloxine staining. We found that the ommatidia organization was completely lost in fly eyes expressing Fus, FusR521G, or Caz (Figure 2K, L, and 2N, respectively), compared to eyes from control flies that had an intact structure (Figure 2J). In contrast, flies expressing FusΔ32 showed a nearly wild-type eye (Figure 2F-G) and had little, if any, retinal degeneration (Figure 2M).

We noted that expressing two copies of Fus or Caz gave rise to more severe eye phenotypes than expressing one copy (compare Figure 2C and 2I to Figure 2B and 2H, respectively). These observations suggest that the toxicity and phenotypes are clearly dependent on Fus/Caz expression levels in the *Drosophila *model system, which supports the hypothesis that the deregulated higher expression of Fus can be toxic. We further found that, in a time course experiment of fly eyes, the overexpression of these proteins produced a progressive eye phenotype, with Caz being the most toxic protein in eyes (Figure 2O-R) and suggesting that the retinal degeneration was progressive with aging. Western blot analysis was performed and confirmed that the proteins were expressed at comparable levels (Additional file 1: Figure S2). We also found that Myc-FusR521C had a similar effect as Myc-FusR521G did (not shown). These findings suggest that Fus/Caz induces toxicity in a dose- and age-dependent manner.

We next systematically characterized the *in vivo *toxicity of various Fus mutations in different tissues using a variety of Gal4 lines. Table S1 summarizes the results of wing, eye, and adult viability results from the above Fus/Caz constructs and two additional mutations. Sequence analysis revealed a highly conserved tyrosine kinase phosphorylation motif (RXXPY) in the C-terminus of Fus/Caz, and mutation of the tyrosine residue in this motif (FusY526F) significantly reduced the *in vivo *toxicity of Fus (Table S1). A consistent but striking finding in these experiments is that the truncation mutation FusΔ32 lacking the C-terminal NLS was largely non-toxic in all lines we tested. We generated a shorter truncation mutation of Fus lacking the RGG-rich domain in the C-terminus (Fus1-453) and found that it was also non-toxic and thus allowed full adult viability (Table S1, data not shown).

### The expression of Fus/Caz in motor neurons causes locomotive impairment

**Figure 3:**
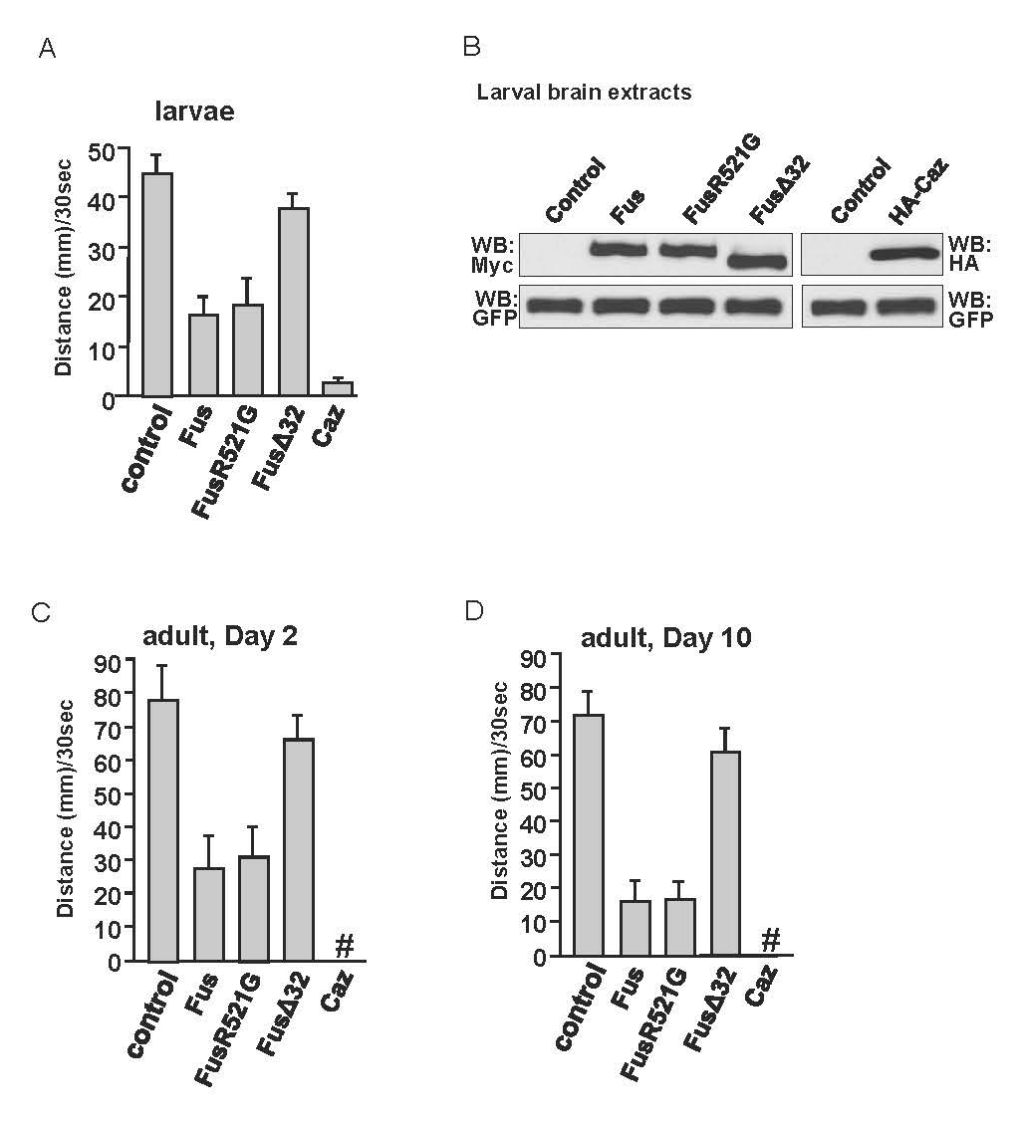
**The locomotive ability affected by the expression of different forms of Fus or Caz**. (A) Expression of Fus/Caz in fly motor neurons impairs the locomotive ability in larvae. A graph of the average moving distance of the third instar larvae traveling over 30 sec is shown here (n = 15 male larvae scored for each group). Each larva was tested 3 times. The *OK371*-Gal4 was used for these experiments. (B) Larvae brains with VNCs from experiments in A were subjected to Western blots with antibodies against the epitope tags to monitor the comparable levels of protein expression. GFP serves as an internal protein expression and loading control. (C-D) Graphs of average climbing distance of the adult flies at Day 2 or Day 10 after emergence. Each adult fly was placed at the bottom of a 2 ml pipette and allowed to climb vertically for 30 s (n = 20 male flies for each group). Each fly was counted 3 times. The height that the flies climbed on the inside wall of the pipette is an indication of their locomotion ability. The *D42*-Gal4 was used for these experiments. The "#" symbol indicates pupal lethality. The larval locomotion was significantly impaired by Fus, FusR521G, and Caz expression, but not by FusΔ32 expression.

We next characterized the locomotive function of the flies overexpressing Fus, Fus mutants, or Caz. We used another motor neuron-specific *OK371*-Gal4, which is expressed earlier than *D42*-Gal4 in the larval stage. When Fus, FusR521G, or Caz was overexpressed through the *OK371*-Gal4 line, the locomotive ability of third instar larvae was markedly impaired (Figure 3A). The average moving distances also correlated with the protein toxicity presented in Table S1. The Caz larvae moved the shortest distance and the FusΔ32 larvae moved the longest distance in a specific period of time among the flies expressing different forms of Fus/Caz (Figure 3A). A comparable level of protein expression in larval brain extracts was confirmed by Western blot (Figure 3B).

We further examined the locomotion of adult flies expressing Fus, Fus mutants, and Caz. We used the other motor neuron-specific Gal4 line, *D42*-Gal4, in the adult movement assays because Fus, FusR521G, and Caz expression by *OK371*-Gal4 caused pupal lethality. We found that the locomotive ability of two-day-old flies was severely impaired by the expression of Fus or FusR521G but not by the expression of FusΔ32 (Figure 3C). Similar results were achieved by analyzing the ten-day-old flies (Figure 3D). In this experiment, the expression of Caz by the *D42*-Gal4 caused pupal lethality, indicating that Caz induced severe toxicity (Figure 3C-D). We also found that the expression of FusR521C produced a similar phenotype as that of FusR521G (data not shown). Taken together, the locomotive defects caused by Fus or Caz suggest that Fus/Caz is involved in neurodegeneration after larval stage.

### Overexpression of Fus/Caz disrupts presynaptic terminals at the neuromuscular junction (NMJ) and causes disorganization of motor neurons in larval ventral nerve cord (VNC)

**Figure 4:**
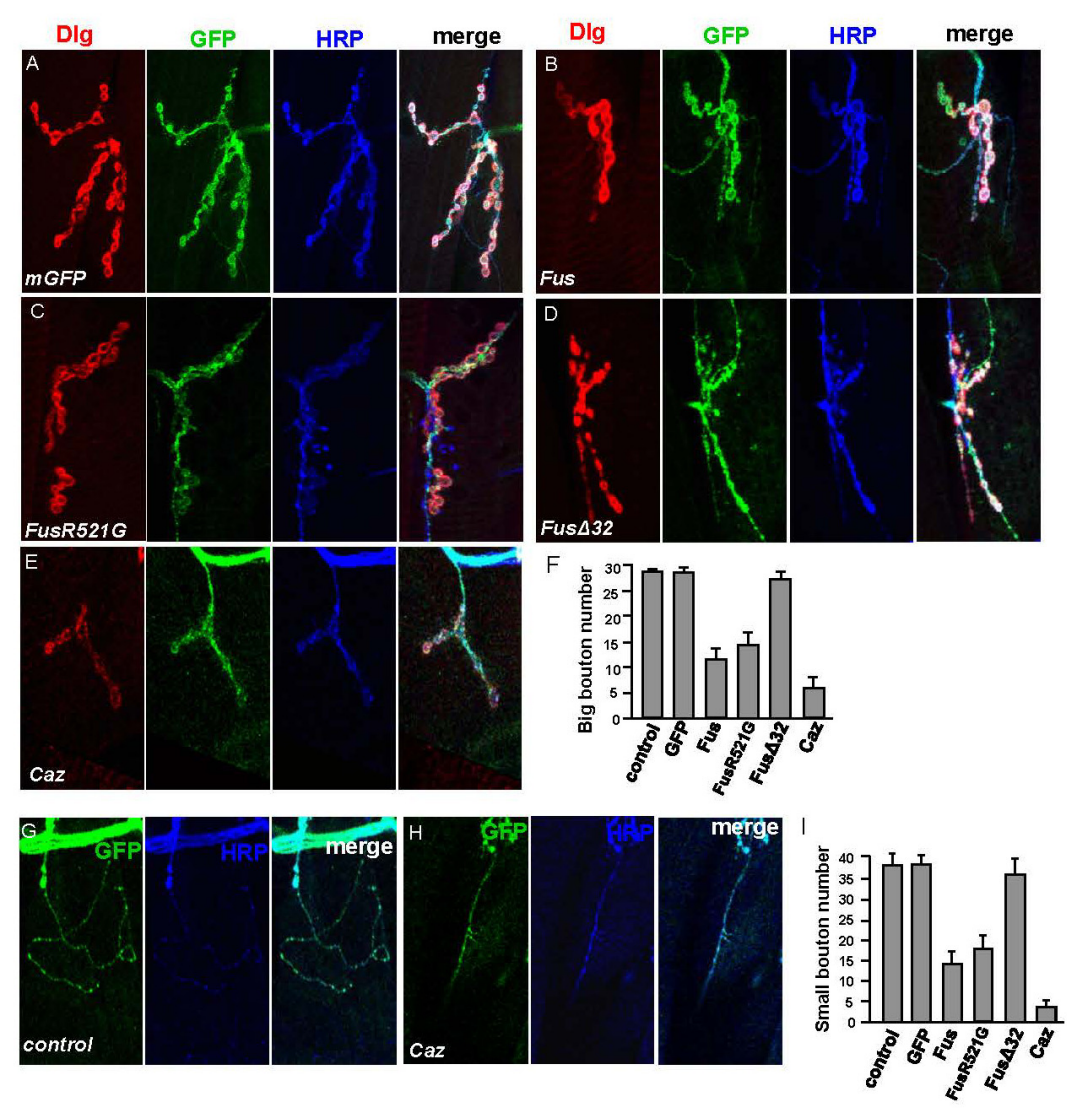
**The overexpression of Fus/Caz causes morphological and functional defects in NMJs**. (A) A representative confocal image (projection over the Z axis) of muscle 6/7 NMJs in the abdominal segment A2 of a third instar larva expressing mGFP by *OK371*-Gal4. The anti-HRP antibody staining labels the neuronal membrane and the anti-Dlg antibody staining marks the subsynaptic reticulum surrounding each bouton. (B-E) NMJs in the abdominal segment A2 from larvae coexpressing mGFP with Fus, FusR521G, FusΔ32, or Caz by *OK371*-Gal4 were stained for Dlg, HRP, and Myc or HA with the appropriate antibodies. Branches and the number of large boutons revealed by Dlg staining were dramatically reduced by Fus, FusR521G, and Caz, but not by FusΔ32 expression. (F) Quantification analysis of the large boutons in the NMJs from ventral longitudinal muscles 6 and 7 of segment A2 (mean ± s.d.; n ≥ 20). *OK371*-Gal4 alone served as control. (G-H) Representative images of axon branches and small boutons, as visualized by both mGFP and HRP staining, at muscle 6/7 NMJs in the abdominal segment A2. (I) Quantification of the small boutons in the NMJs from ventral segment A2 (mean ± s.d.; n ≥ 20). *OK371*-Gal4 alone served as control.

To gain more insights into the disease etiology, we analyzed the morphology of motor neuronal presynaptic terminals at the NMJ in flies. Third instar larval muscle fillets were dissected and double-labeled with an anti-HRP antibody to visualize the neuronal membrane and an anti-Dlg antibody to mark the subsynaptic reticulum surrounding each bouton. The expression of a membrane GFP (mGFP) by *OK371*-Gal4 was used to mark the motor neurons and did not have any effect on bouton formation at NMJ (Figure 4A, F and 4I). Morphological abnormalities were found in larvae co-expressing mGFP with either Fus or Caz by *OK371*-Gal4. As shown in Figure 4, the overexpression of Fus substantially reduced the area (Figure 4B) and the number of both large and small boutons (Figure 4F and 4I). Compared to Fus, expression of Caz had more severe NMJ defects (Figure 4E, F, H, and 4I). Interestingly, overexpression of FusΔ32 induced little, if any, disruption in the presynaptic terminals (Figure 4D, F, and 4I). These findings are consistent with the locomotive deficiency observed in the animals (Figure 3C-D), suggesting that the abnormal NMJs in flies expressing Fus are responsible for the impaired motor function. The results are also consistent with the toxicity of Fus/Caz observed in other tissues (Additional file 1: Table S1).

**Figure 5:**
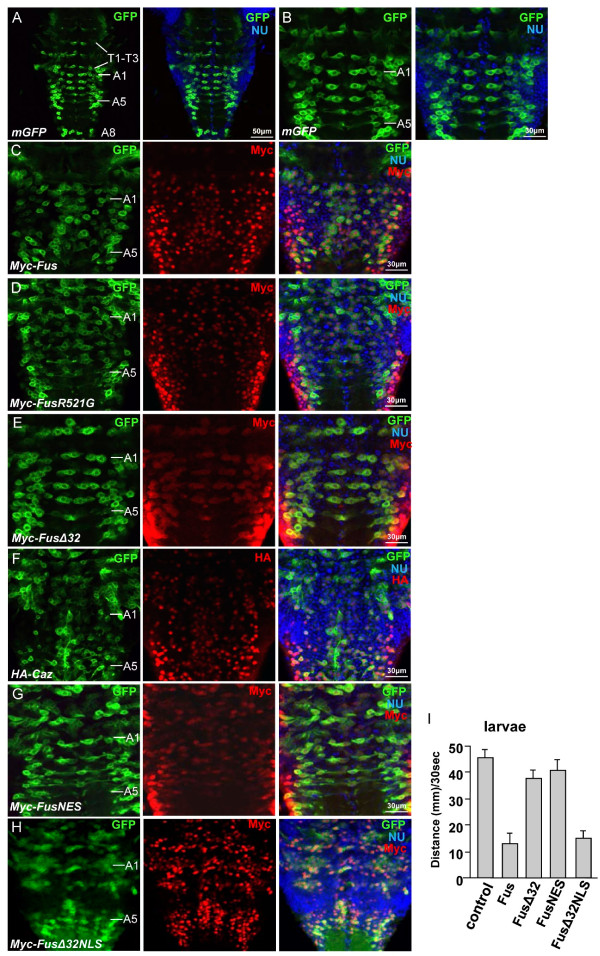
**The expression of Fus/Caz disrupts the arrangement of motor neurons in the VNC**. (A) A VNC from the third instar larva expressing mGFP by *OK371*-Gal4 was stained with nuclear dye Hoechst 33342 (blue NU signal). The GFP signal indicates intact motor neurons. T1-T3 indicates the three thoracic neuromeres and A1-A8 indicates the eight abdominal neuromeres. (B) Shown here are the well-organized segmental motor neurons of the abdominal neuromeres A1-A5. The organization of motor neurons is used as a model to examine the neuronal toxicity of Fus/Caz in this study. (C-E) VNCs from third instar larvae co-expressing mGFP with Myc-tagged Fus, FusR521G, or FusΔ32 by *OK371*-Gal4 were stained with an anti-Myc antibody and Hoechst. Fus and FusR521G disrupted the organization of motor neurons in A1-A5 neuromeres, but FusΔ32 did not. (F) A VNC from larva coexpressing mGFP with HA-Caz was stained with an anti-HA antibody and Hoechst 33342. The expression of Caz severely disrupted the arrangement of motor neurons in A1-A5 neuromeres. (G-H) VNCs from larvae coexpressing mGFP with Myc-FusNES or Myc-FusΔ32NLS were stained with the anti-Myc antibody and Hoechst 33342. (I) A graph of the average moving distance of the third instar larvae expressing the indicated proteins and traveling over 30 sec (n = 15 male larvae scored for each group). Each larva was tested 3 times. The *OK371*-Gal4 was used as the driver.

To further elucidate whether Fus overexpression caused any defects in motor neuron cell bodies, we next examined the motor neurons in larval ventral nerve cord (VNC) from late third instar larvae expressing different Fus/Caz proteins by the *OK371*-Gal4. Motor neurons in the ventral clusters are well-organized in the larval VNC in wild-type flies (Figure 5A). In addition, longitudinal motor neurons, which ensheath the cortex of the VNC, are logitudinally connected, and the segmental nerve of the abdominal neuromeres A1-A5 are well-organized in wild-type flies (Figure 5B). As expected, we found that the *OK371*-Gal4-mediated expression of Fus, FusR521G, or Caz induced disorganized motor neurons in A1-A5 of the larval VNC (Figure 5C, D, and F). In contrast, the overexpression of FusΔ32 again had no effect on the organization of neuromeres A1-A5 (Figure 5E), which was consistent with the finding that this truncation mutant had no toxicity in motor neurons (Figures 3 and 4).

To solidify the relationship between nuclear localization and toxicity, we constructed Myc-tagged FusNES with a typical nuclear export signal (NES) fused in frame to the C-terminus of FUS (Myc-FusNES) and Myc-tagged chimeric FusΔ32NLS with the NLS of another RNA binding protein hnRNP D fused to the C-terminus of FusΔ32 (Myc-FusΔ32NLS). We then generated transgenic lines at the 75B1 - VK5 attP locus. Expression of FusNES by the *OK371*-Gal4 had markedly less effect in larva VNC (Figure 5G) and did not induce locomotive defects in the third instar larvae (Figure 5I). Thus, both FusΔ32 and FusNES were largely non-toxic in a similar fashion. In contrast, expression of FusΔ32NLS induced disorganization of motor neurons in VNC (Figure 5H) and caused locomotive defects at a comparable level as in flies expressing Fus (Figure 5I). We also found that FusNES did not cause any phenotype in the eyes or NMJs when driven by *GMR*-Gal4 or *OK371*-Gal4, whereas FusΔ32NLS did (Table S1).

We examined the subcellular localization of Fus/Caz proteins using confocal microscopy. Consistent with our previous findings in mammalian cells [19], we found that Fus, Caz, and FusΔ32NLS were largely localized in the nuclei of motor neurons in the VNC (Additional file 1: Figures S2A and S2D). There was a small increase in cytoplasmic staining of FusR521G (Additional file 1: Figure S2B), whereas FusΔ32 and FusNES were largely cytosolic (Additional file 1: Figure S2C and S2E). Taken together, the data from all tissues examined (Figures 1, 2, 3, 4) suggest that the toxicity of Fus/Caz in flies is likely due to, at least in part, the abnormal function caused by the nuclear fraction of Fus/Caz protein.

### The expression of Fus/Caz induces apoptosis in motor neurons

**Figure 6:**
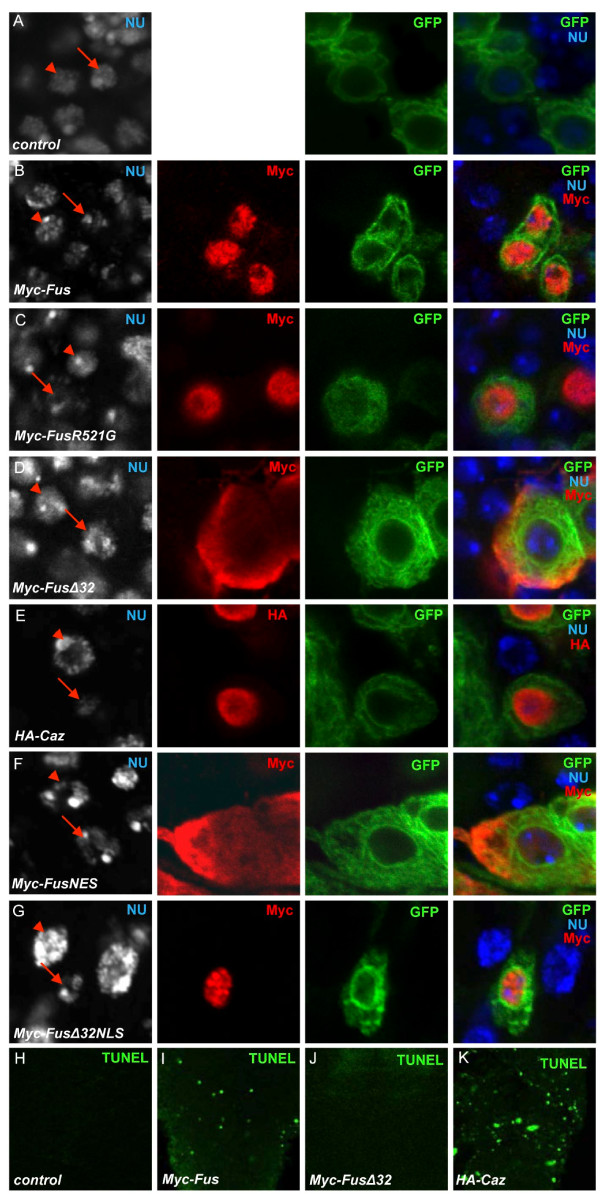
**Neurodegeneration induced by Fus/Caz overexpression occurs due to apoptosis**. (A) A large magnification of the VNC from a third instar larva expressing mGFP by *OK371*-Gal4 shows GFP-labeled motor neurons and adjacent non-motor neuronal cells. Arrows in A indicate the nuclear structure of motor neuronal cells and arrowheads indicate the nuclear structure of adjacent cells. (B-G) VNCs from larvae coexpressing mGFP with Myc-Fus, Myc-FusR521G, HA-Caz, Myc-FusNES, or Myc-FusΔ32NLS were stained with the indicated antibodies and the Hoechst 33342 dye to label the nuclear structures. Arrows in B, C, E, and G indicate the decreased nuclear staining and disrupted nuclear structure by the expression of Fus, FusR521G, Caz, or FusΔ32NLS in the nuclei. Arrows in D and F indicate the unaffected nuclear structures. The arrowheads indicate the nuclear staining of normal adjacent cells, which serves as a control. (H-K) TUNEL assays were performed on the VNC from the larvae of *OK371*-Gal4 (control) or the larvae expressing Fus, FusΔ32, or Caz by *OK371*-Gal4. Apoptotic cell death is measured by the TUNEL signal (green).

In the above experiment, we observed an abnormal nuclear structure in motor neurons overexpressing Fus/Caz when the cells were stained with Hoechst 33342 nuclear dye. Motor neuron cells expressing the mGFP alone showed a normal even nuclear staining (arrow in Figure 6A), which was almost identical to the structure of the adjacent non-motor neuronal cells (arrowhead in Figure 6A). Surprisingly, the overexpression of Fus or Caz with mGFP resulted in decreased nuclear staining reminiscent of apoptotic bodies in the GFP-positive motor neurons (arrows in Figure 6B and 6E). In contrast, the nuclear staining was largely uniform in the adjacent non-Fus/Caz-expressing cells (arrowheads in Figure 6B and 6E). Consistent with the earlier data, the expression of FusR521G gave rise to a similar effect to that of Fus (arrow in Figure 6C). The expression of FusΔ32 and FusNES gave rise to normal nuclear staining in motor neurons (arrows in Figures 6D and 6E), whereas FusΔ32NLS induced abnormal nuclear staining (arrow in Figure 6G). These data again suggest that the toxicity of Fus/Caz is caused, at least in part, by the protein localized in the nucleus.

We next performed the terminal deoxynucleotodyl transferase dUTP nick end labeling (TUNEL) assay to examine whether the expression of Fus/Caz could induce apoptosis in the VNC. We did not detect any TUNEL-positive cells in control third instar larval VNC (Figure 6H). However, Fus expression in VNCs led to approximately 20-30 TUNEL-positive cells in the same larval stage (Figure 6I), suggesting that overexpression of Fus induced neuronal apoptosis in the VNC. In contrast, the overexpression of FusΔ32 or FusNES did not give rise to TUNEL-positive cells (Figure 6J, not shown), which was consistent with the finding that FusΔ32 and FusNES induced little disorganization of motor neurons in the VNC. In addition, we found that the overexpression of Caz induced approximately 40-50 TUNEL-positive cells in the VNC (Figure 6K), which could explain why the overexpression of Caz at an equivalent level as Fus led to a more severe toxicity. Our results suggest that the neurodegeneration induced by Fus/Caz overexpression occurs due to apoptosis in the *Drosophila *VNC.

### The phenotypes of *caz *deletion mutant *Drosophila*

**Figure 7:**
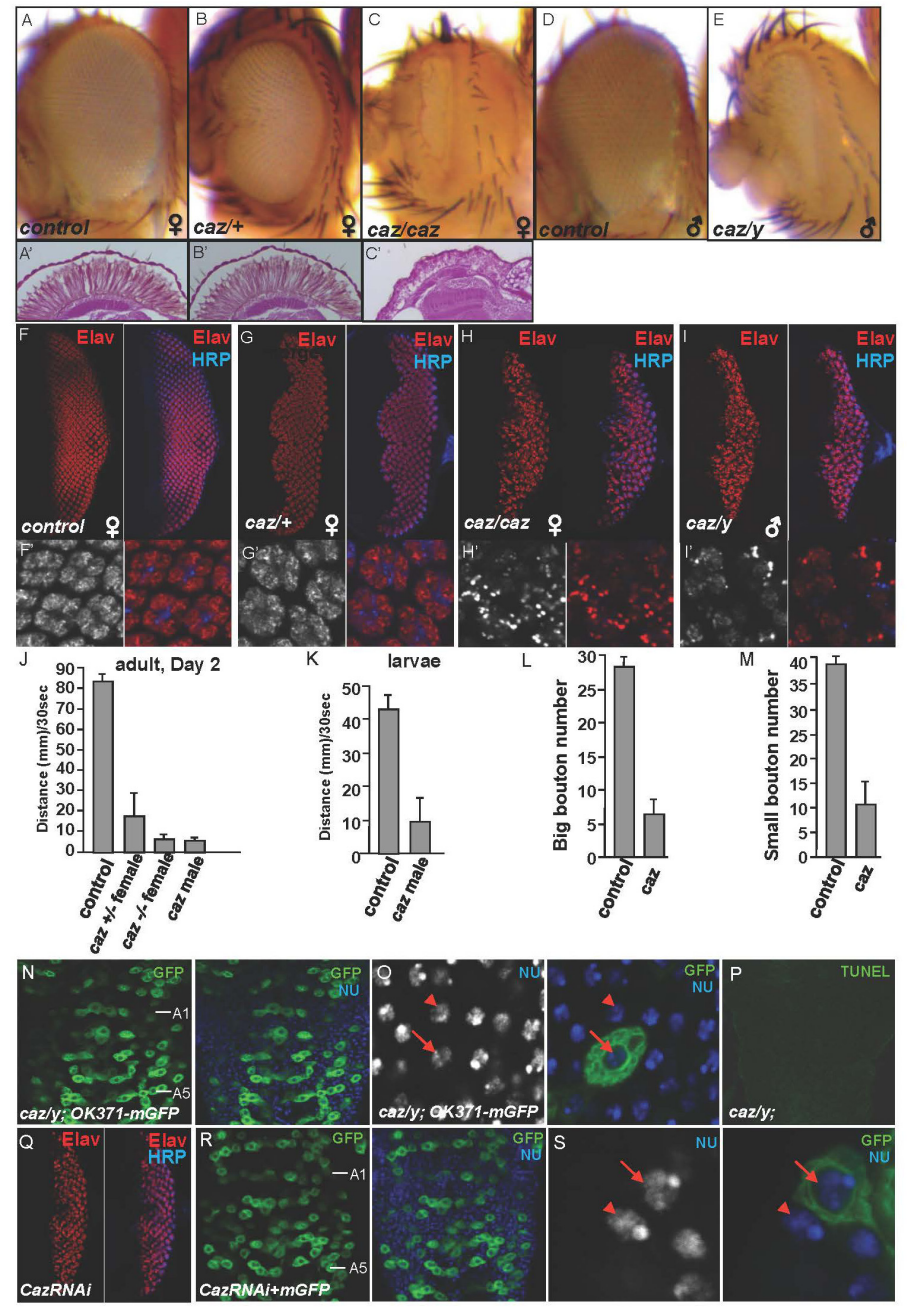
**The loss of *caz *leads to neuron functional defects in *Drosophila***. (A-A') An eye from *yw *female flies showing wild-type ommatidial and rhabdomere structure. (B-B') An eye from *caz *heterozygous female flies shows the balancer marker and intact ommatidial and rhabdomere structure. (C-C') An eye from *caz *homozygous female fly shows the loss of ommatidia and the disrupted rhabdomere structure. (D) An eye from *yw *male fly showing normal ommatidial. (E) An eye from *caz *mutant male showing the loss of ommatidia. (F-F') A wild-type eye imaginal disc from the third instar larvae was stained for Elav and HRP to show the well-developed and well-organized structure of the ommatidia units. (G-H') Eye discs from either *caz *heterozygous or homozygous female third instar larvae were immunostained for Elav and HRP. (I-I') An eye disc from *caz *male larva was stained for Elav and HRP. (J) A graph of the average climbing distance of *caz *mutant adult flies at Day 2 after emergence. The same method was used as in Figure 4. N = 20 flies for each genotypic group. (K) A graph of the average moving distance of the third instar larvae of *caz *males traveling over 30 s (n = 15 male larvae scored for each group). Each larva was tested 3 times. (L-M) Quantification analysis of the large and small boutons in the NMJs from ventral longitudinal muscles 6 and 7 of segment A2 in *caz *mutant male larvae (mean ± s.d.; n ≥ 20). (N) A VNC from a *caz *mutant male fly expressing mGFP by *OK371*-Gal4 shows the disrupted motor neuron organization in A1-A5 neuromeres. (O) A large magnification image from N. The arrow indicates the motor neuron nuclear staining, which is similar to the nuclear staining of an adjacent cell (arrowhead). (P) TUNEL assay was performed on VNC from the larvae of a *caz/+ *female, *caz/caz *female, and *caz *male. A representative TUNEL image from the VNC of a *caz *male is shown here. None of the *caz *mutant larvae showed TUNEL-positive cells. (Q) An eye imaginal disc expressing Dicer and CazRNAi by *GMR*-Gal4 was stained for Elav and HRP. (R) A VNC from a third instar larva coexpressing mGFP with Dicer and CazRNAi shows the disorganization of motor neurons in A1-A5 neuromeres. (S) A large magnification image from R. The arrow indicates the motor neuron nuclear staining, which is similar to the nuclear staining of an adjacent cell (arrowhead).

Our initial study of *caz *inactivation in flies using the RNAi lines from the Vienna *Drosophila *RNAi Center (VDRC) found that CazRNAi caused a defective morphology in the wing (Figure 1F) and reduced the viability of adult flies (Figure 1G-H). Next, we explored the neuronal function of Caz by analyzing *caz *mutant phenotypes. A deficiency line *DF(1)BSC759*, which harbors a deletion in *caz *and other genes upstream of *caz*, was first used. *DF(1)BSC759 *heterozygous females exhibited wild-type eyes with the balancer marker (Figure 7B), but *DF(1)BSC759 *homozygous females were lethal at the pupa stage and the adult escapers exhibited loss of ommatidia in the eyes (Figure 7C and Additional file 1: Figure S3C). *DF(1)BSC759 *males also showed a similar eye phenotype (Figure 7E and Additional file 1: Figure S3E). These data suggest that the lack of Caz disrupts the formation of *Drosophila *ommatidia. Another deficiency line *DF(1)ED7355*, which retains the wild-type *caz *gene but lacks a fragment upstream of *caz*, was able to suppress the eye phenotypes in the complementary test. The results suggest that the phenotype in *DF(1)BSC759 *was caused by the loss of *caz *but not other genes. We also examined the other *caz *mutant allele *caz1 *[27] and found that *caz1 *mutant had similar phenotypes as *DF(1)BSC759*.

We next examined how *caz *deletion caused the degenerative phenotypes in eyes as seen in the *caz *homozygous flies (Figure 7C', compared to Figures 7A' and 7B'). An anti-HRP antibody was used to detect the photoreceptor neurons and an anti-Elav antibody to detect the pan neurons in third instar larvae. The Elav signal was evenly distributed in the well-organized ommatidia units of eye discs in the wild-type (Figure 7F-F') and *caz *heterozygous female (Figure 7G-G') flies. In contrast, the eye discs from *caz*/*caz *homozygous female (Figure 7H-H') and *caz*/*y *male (Figure 7I-I') flies showed a substantially decreased Elav staining with abnormal puncta in disorganized ommatidia. In addition, Caz knockdown by RNAi also disrupted ommatidia formation (Figure 7Q). These data suggest that Caz is required for maintaining normal function of the neurons in the eye.

We next assessed the function of Caz in *Drosophila *motor neurons. Loss of *caz *in motor neurons significantly impaired the locomotive ability of adult flies of both males and females (Figure 7J) and shortened the lifespan of adult flies (data not shown). Male *caz *mutant larvae showed severe reduction in locomotive activity (Figure 7K). Examination of the NMJs in the male mutant larvae revealed a severe decrease in numbers for both big and small boutons in the NMJs (Figure 7L-M). The eye phenotype was fully rescued by expressing wild-type Fus or Caz (data not shown), which is consistent with the findings in fly locomotive function rescue from a previous study [27]. These results suggest that Caz is required for the function of photoreceptor neurons as well as motor neurons.

We further assessed the organization of the motor neurons in VNC in *caz *mutant flies as we did in the Fus/Caz overexpressing flies. We found that *caz *mutant male flies exhibited disorganized motor neurons in neuromeres A1-A5 of the VNC (Figure 7N), a phenotype similar to that caused by the overexpression of Fus/Caz (Figure 5C and 5F). However, unlike the Fus/Caz overexpression flies, loss of *caz *did not cause any decrease in nuclear staining by Hoechst 33342 (arrow in Figure 7O). Mutating *caz *in the motor neurons also did not produce any TUNEL-positive cells in the VNC (Figure 7P). These results suggest that the loss of *caz *did not promote cell death of motor neurons although it disrupted NMJs and impaired locomotive function in the *caz *mutant flies. Consistent with these results, Caz RNAi also interrupted the arrangement of motor neurons in VNCs (Figure 7R), but did not cause any decrease in nuclear staining (Additional file 1: Figure S7) or any TUNEL-positive cells (not shown). Collectively, these data suggest that the mechanism underlying the phenotypes in the *caz *mutant line is different from that in the Fus/Caz overexpression flies.

## Discussion

The vast majority of the molecules critical to neuronal function are conserved between *Drosophila *and more complex vertebrate systems, thus *Drosophila *is a powerful model to study neurodegenerative diseases including ALS. In this study, we showed that the overexpression of human Fus as well as the *Drosophila *homolog Caz caused neuronal toxicity *in vivo *in all Gal4 lines that we tested, including ubiquitous expression (*act5C*-Gal4), wings (*MS1096*-Gal4), eyes (*GMR*-Gal4), neurons (*Elav*-Gal4) and motor neurons (*D42- *and *OK371*-Gal4). Since Fus/Caz protein was expressed at late larval or pupal stages and the degenerative phenotypes were progressive with aging, the Fus/Caz overexpressing flies are a reasonably good model to study the relevance of Fus in motor neuron degeneration in ALS. Furthermore, we provide evidence that both loss of Caz and overexpression of Fus/Caz can induce phenotypic defects although the underlying cellular mechanisms are likely to be different in these two models.

### Nuclear toxicity of Fus/Caz in the absence of cytoplasmic inclusions

It has been recently shown that the overexpression of Fus in flies leads to neuronal toxicity [25-27]. However, it is unknown whether overexpression of *Drosophila *Caz can cause toxicity. In this study, overexpression of fly Caz caused similar or even more severe toxicity in all experiments compared to overexpression of human Fus (Figures 1, 2, 3, 4). This is reminiscent of the studies of another ALS-implicated RNA binding protein TDP-43 that the overexpression of TDP-43 was shown to be toxic in yeast [33], *Drosophila *[23,24,34,35], *C. elegans *[36], and mouse [37,38]. It is noted that protein inclusions of wild-type Fus have also been reported in human ALS patients [7,8]. These findings underscore the importance of understanding the underlying molecular mechanisms of Fus in ALS.

Our group and others have shown that the last 32 amino acids of the protein contain an NLS that is both essential and sufficient for nuclear targeting. It has also been shown that the ALS-related mutations in Fus increased the cytoplasmic localization of the protein and promoted the co-localization of Fus with stress granules in the cytosol [19-21]. However, the cytoplasmic accumulation may not be essential or sufficient for the toxicity observed for the protein [39,40]. Thus, the link between toxicity and subcellular localization of Fus needs to be characterized *in vivo*.

A novel finding of this study is that Fus nuclear localization is required for its toxicity in *Drosophila*. The overexpression of FusΔ32 at comparable levels consistently showed little or no toxicity in all tissues. The *in vivo *subcellular localization of wild-type Fus, R521G mutant and FusΔ32 was confirmed to be consistent with the findings in cell culture, i.e. FusΔ32 was largely diffused outside of the nucleus. Moreover, the fusion of an NES at the C-terminus of the full-length wild-type Fus dramatically reduced toxicity *in vivo *(Figures 5G, I and 6F). FusNES was also diffused in the motor neurons (Additional file 1: Figure S2), thus the data from both FusNES and FusΔ32 suggest that the nuclear localization is critical to the toxicity. Furthermore, the chimeric protein FusΔ32NLS with an exogenous NLS was localized in the nucleus (Additional file 1: Figure S2) and produced similar toxicity as wild-type Fus (Figure 5H, I, and 6G). All these results consistently suggest that the nuclear localization is required for Fus toxicity.

This surprising result differs from the common presumption that the toxicity is caused by the cytoplasmic accumulation of Fus. Our study is the first one characterizing transgenic flies expressing truncated Fus mutant with a primarily cytoplasmic localization. We further manipulated the subcellular localization by using a typical NES and the other different NLS. All experiments consistently support the conclusion. Interestingly, another study showed that mutation in the NLS of TDP-43 relieved the toxicity in *C. elegans *[36], supporting the conclusion of our study. However, the study by Lanson *et al*. employed another approach by deleting a postulated NES within the RNA recognition motif of Fus and showed reduced toxicity [25]. It should be noted that Lanson *et al*. did not characterize the NES or the subcellular localization of the NES deletion mutant. Moreover, the postulated NES is within the RNA binding domain in Fus, therefore such deletion may cause a disruption of the RNA binding properties of Fus. Thus, the mechanism of the reduced toxicity of that particular NES deletion mutant needs to be better elucidated. We are confident in our conclusion since the results using three different approaches (FusΔ32, FusNES, and FusΔ32NLS) consistently support the requirement of nuclear localization for Fus toxicity.

Another critical difference between this study and the study by Lanson *et al*. is how the transgenic flies were generated. The site-specific transgenic approach using the integrase-mediated insertion at the specific attP locus allowed us to ensure equal expression of the Fus proteins without positional effects. We think that the discrepancy is likely due to the expression level of the protein. This likely explains the discrepancy regarding the toxicity of the Fus transgene in different studies. The R521G mutation produced comparable levels of toxicity as wild-type Fus whereas the other study showed more severe toxicity in flies expressing R521H or R521C [25]. The experiments in our study are more likely to produce comparable expression levels of the Fus transgene since transgene insertion was random in other studies.

Disruption of muscle 6/7 NMJs was observed in the Fus transgenic flies in this study. It is important to note that NMJ vulnerability occurs selectively in different subgroups of motor neurons or muscles. The diversity of subtypes of motor neurons and muscles and their susceptibility are well documented in ALS [41]. We examined the muscle 6/7 NMJs in the abdominal segment A2 of the third instar larvae, which is widely used in *Drosophila *studies. In addition, we used GFP as a marker to monitor the target protein expression. Our results showed the expression of Fus/Caz in the disrupted NMJs. Studies characterizing NMJs in other muscles may obtain different results, for example another study examined muscle 4, segment A2 and 3 and did not observe reduction of synaptic boutons in Fus overexpressing flies [25].

Notably, neither wild-type Fus/Caz nor the ALS mutants of Fus showed cytoplasmic inclusions in transgenic flies where severe toxicity was demonstrated in this study. The results suggest that the cytoplasmic inclusions are not essential to Fus toxicity although they have been prominently observed in cell culture systems and in human patient tissues. It has long been proposed that soluble oligomers may be the culprit species causing neurodegenerative disease. A recent study showed that aberrant high molecular weight complex of TDP-43 was toxic, although the nature of the species was still unknown [42]. A recent study also showed that Fus toxicity could be suppressed without eliminating protein inclusions in yeast [40], suggesting that protein inclusions might be a non-toxic and non-essential feature of the disease.

Although the results in this study consistently suggest that sufficient steady-state levels of Fus/Caz are required for the toxicity and neurodegenerative phenotypes, it is noted that Fus/Caz undergoes dynamic trafficking in and out of the nucleus in live cells. It remains possible that Fus/Caz with steady-state nuclear localization could produce detrimental effect while it is temporarily outside of the nucleus. Alternatively, the minute amount of Fus/Caz in the cytoplasm that cannot be detected by confocal microscope could also produce cytosolic toxicity. We believe that these two are remote possibilities, however additional studies using more sophisticated approaches are needed to test them in the future. Moreover, the exact nature of Fus toxicity in the nucleus also remains to be determined in future studies.

### The phenotypes of *caz *deletion in *Drosophila*

Deletion of a specific gene provides insight into understanding the *in vivo *function of the protein. By taking advantage of the *Drosophila *model, we found that endogenous Caz is required for normal functions of the neurons (Figure 7). The deficiency of *caz *in flies induced a strong eye phenotype with disrupted ommatidia structure (Figure 7A-I) and caused defects in the locomotive function in larvae (Figure 7K) and adult flies (Figure 7J). Consistently, *caz1 *mutant flies exhibited decreased adult viability and diminished locomotive activity [27]. These phenotypes were actually similar to those caused by Fus/Caz overexpression. It would be interesting to examine whether some cases of ALS disease may be caused by Fus loss-of-function in patients.

Given the motor deficiencies of Fus overexpression and mutant *caz *flies, the presynaptic structure in NMJ was examined. As expected, the number of boutons was significantly reduced in the larvae overexpressing Fus (Figure 4) as well as in the larvae lacking *caz *(Figure 7L-M). Thus the disruption of NMJ was evidently the cause of the locomotive defects. Therefore, the Fus/Caz overexpression and loss of *caz *obviously caused similar disruption of presynaptic terminals in NMJs. It is likely that Caz is required for neuronal function and therefore the lack of Fus/Caz perturbs the motor neuron distal terminus and causes NMJ disruption.

### Different molecular mechanisms for degenerative phenotypes in Fus overexpression and *caz *deletion *Drosophila*

By examining the nuclear structure and monitoring apoptosis of cells in the VNC, we found that overexpression of Fus/Caz induced apoptotic cell death (Figure 6). However, neither the *caz *deficiency (*BSC759*) nor the *caz1 *mutation led to apoptosis (Figure 7P and 7S, data not shown). Despite the critical difference, both Fus/Caz overexpression and *caz *deletion disrupted the NMJs. This raises the question of where the toxicity originates, either from the death of neuronal cell bodies or disruption of the NMJs.

Studies in the SOD1 mutant-mediated familial ALS have suggested an axon die-back model in which perturbation at the distal NMJs occurs prior to the death of motor neurons [41]. The Fus/Caz overexpression phenotypes support this model, but the time sequence of the NMJ disruption and motor neuron death has not been distinguished in these flies as yet. Moreover, both the *caz *deficiency and *caz1 *mutant flies strongly support this model since loss of Fus/Caz caused NMJ disruption and motor function deficiency without the presence of motor neuronal cell death. The findings in our study support two potential mechanisms for the Fus/Caz mediated neurotoxicity: neuronal apoptosis and NMJ perturbation. These two mechanisms are not necessarily mutually exclusive, and in fact may occur simultaneously. Further studies exploring of the in-depth mechanisms by which Fus/Caz causes alterations in RNA processing and nuclear toxicity are currently ongoing. Many laboratories in the field also use various models, including yeast [39,40,43,44] and *Drosophila *[25-27], to investigate the role of Fus in RNA metabolism and the genetic interactions between Fus and other RNA processing proteins. The overexpression and deletion *Drosophila *models in this study provide valuable insight into the etiology of ALS and potentially other degenerative diseases in which RNA processing proteins are implicated.

## Conclusions

We conclude from our study that the expression of Fus can cause nuclear toxicity, induce apoptosis and facilitate NMJ perturbation in the transgenic *Drosophila *model of ALS. Generation and characterization of the *Drosophila *model of Fus-mediated ALS have revealed several novel findings and provided new insights into the disease etiology. There are three major novel findings in this study: (1) the C-terminus of Fus is essential for its *in vivo *toxicity, suggesting that Fus toxicity originates from the nuclei instead of the cytoplasm; (2) both the overexpression of Fus/Caz and the deletion of *caz *caused similar deficiency in locomotive function and disruption in NMJs; (3) the overexpression of Fus/Caz induced apoptosis in motor neuron cell bodies, whereas no apoptosis was observed in the motor neurons of the *caz *mutant flies. In addition to the novel findings, we have also developed assays to investigate the functions of Fus/Caz in the *Drosophila *VNC. The novel neuronal functions of Caz that we have uncovered here are likely to be conserved in other species.

## Methods

### Constructs, mutants, and transgenes

UAST-Myc-Fus was generated by subcloning the cDNA fragment of human Fus into the attB-UAST-5xMyc vector. Amino acid substitutions of R521G, R521C, and Y526F were generated by PCR-based site-directed mutagenesis. FusΔ32 and Fus1-453 were generated by truncation at F494 and G453, respectively. FusR521G, FusR521C, FusY526F, Fus1-453, and FusΔ32 were subcloned into attB-UAST-5xMyc using the same approach as used for Fus. To generate FusNES, the typical NES sequence (NINELALKFAGLDI) [45] was fused in frame with the C-terminus of Myc-Fus. To generate FusΔ32NLS, the NLS sequence (YGDYSNQQSGYGKVSRRGGHQNSYKPY) from hnRNP D [46] was fused in frame with FusΔ32. To generate HA-tagged Caz (HA-Caz), we obtained cDNA clone (LD22761) from the *Drosophila *Genome Resource Center (DGRC, USA) and subcloned the full-length Caz cDNA into attB-UAST-HA vector. To ensure that the proteins were expressed at the same level without a positional effect, the PhiC31 integration system was used to integrate Fus and Caz transgenes into the 75B1 attP locus in the fly genome [30,31]. The Caz RNAi line, v100291, and the *UAS-Dicer *lines were obtained from the VDRC (Austria). *DF(1)BSC759 *and *DF(1)ED7355 *were obtained from the Bloomington *Drosophila *Stock Center (USA). *caz1 *mutant flies carry deletion Df[1]383 and a genomic transgene for CG32576, which is supposed to be a *caz *null [27]. *MS1096*-Gal4; *D42*-Gal4, *Elav-*Gal4, *act5C*-Gal4, *GMR*-Gal4, *OK371*-Gal4, *UAS-Dicer*, and *yw *have been previously described (Flybase) [31].

### Adult eye histology, immunoblot analysis, and eye imaginal disc immunostaining

To analyze the internal structure of adult eyes, the heads of flies from specific genotypes at 4 day-after-eclosion (DAE) were fixed in Bouin's fixative solution (Cat# HT10132, Sigma, USA) for 48 h at room temperature on a shaker at low speed, incubated 24 h in 50 mM Tris/150 mM NaCl, and then embedded in paraffin. Serial sections (5 μm thickness) were taken through the entire head, stained with Eosin (Cat# S176, Poly Scientific, USA), and examined under a Leica DM750 microscope (Leica, Germany). Western blot was performed to confirm comparable levels of protein expression. Fifteen heads from adults expressing different Fus or Caz transgenes by *GMR*-Gal4 were dissected and subjected to direct Western blot with mouse anti-Myc (9E10, 1:5000, Santa Cruz, USA) and anti-β-tubulin (1:5000, Developmental Studies Hybridoma Bank; DSHB, USA). A standard protocol was used for the eye imaginal disc immunostaining assay. Eye discs were dissected, fixed in 4% paraformaldehyde, washed in phosphate-buffered saline with 0.3% Triton-X100 (PBT), and then stained with mouse anti-Elav (1:50, DSHB) for 2 h at room temperature. After washing with PBT, the eye discs were stained with goat anti-mouse Rhodamin (1:500, Jackson ImmunoResearch, USA) and rabbit anti-Horseradish Peroxidase (HRP) conjugated to Cy5 (1:500, Jackson ImmunoResearch) for 2 hours at room temperature.

### Dissection, immunostaining, and analysis of NMJs from third instar larvae

Late third instar larvae were dissected, fixed in 4% paraformaldehyde, washed in PBT, and stained with an anti-Discs-large (Dlg) antibody (1:100, DSHB) at room temperature for 2 h. After washing with PBT, the discs were stained with goat anti-mouse Rhodamin (1:500, Jackson ImmunoResearch) and rabbit anti-HRP antibody conjugated to Cy5 (Jackson ImmunoResearch, 1:500). Samples were then mounted and viewed with an Olympus confocal microscope. Z-scan sections of the NMJs from ventral longitudinal muscles 6 and 7 at segment A2 were taken at 1.0 μm intervals by confocal imaging (Olympus Fluoview, Ver.1.7c) to visualize the HRP-positive neuronal membrane and the Dlg-positive large boutons. The number of large and small boutons was also quantified after intensity projection over the Z axis.

### Immunostaining and TUNEL assay of the VNCs from third instar larvae

Fly larval VNCs with brains from specific genotypes were dissected, fixed in 4% paraformaldehyde, and immunostained with mouse anti-Myc (9E10, 1:50, Santa Cruz) or anti-HA (F-7, 1:150, Santa Cruz) for 2 h at room temperature. After washing with PBT, the tissues were stained with goat anti-mouse Rhodamin (1:500, Jackson ImmunoResearch). Hoechst dye 33342 (1 μg/ml, Invitrogen, USA) was added with the secondary antibody staining. For TUNEL staining, samples were pretreated with 3 μg/ml Proteinase K for 10 min at room temperature, fixed in 4% paraformaldehyde, and permeabilized in PBT with 0.1% sodium citrate. Samples were then treated with TUNEL (In Situ Cell Death Detection Kit) with green fluorescence (Roche, Cat# 11684795910) followed by imaging analysis with the Olympus confocal microscope.

Western blot was performed to confirm protein expression at a comparable level. Five brains containing VNCs from larvae expressing Fus variants or Caz by *OK371*-Gal4 were dissected and lysed in regular Schneider S2 cell lysis buffer, and then subjected to direct Western blot with mouse anti-Myc (9E10, 1:5000, Santa Cruz), anti-HA (F-7, 1:1500, Santa Cruz), or anti-GFP (1:1000, Millipore, USA).

### Larval movement and adult climbing assays

Third instar larvae bearing specific genotypes were subjected to motor function analysis on 100 mm agar plates. Individual larvae were placed on plates and stimulated to move with a needle. The distance they traveled over the next 30 s was traced and measured. Each individual larva was counted 3 times and 20 larvae were tested for each group. The climbing ability of adult flies with different genotypes was examined in empty 2 ml pipettes vertically. Flies were first placed at the bottom of the pipette and the climbing distances were scored over 30 s. Each fly was recorded 3 times and 20 flies in each group were tested.

## Abbreviations

ALS: Amyotrophic lateral sclerosis; Fus: Fused in sarcoma; Caz: Cabeza; VNC: Ventral nerve cord; NLS: Nuclear localization sequence; NES: Nuclear export signal; SOD1: Cu/Zn superoxide dismutase; NMJ: Neuromuscular junction.

## Competing interests

The authors declare that they have no competing interests.

## Authors' contributions

RX, YL, LY, JG performed the experiments. HZ and JJ analyzed the results, designed the study, and wrote the manuscript. All authors read and approved the final manuscript.

## Supplementary Material

Additional file 1 **Figure S1 The *caz *gene encodes the *Drosophila *homolog of Fus**. A sequence alignment between Caz, mouse Fus, and human Fus is shown here. The SYQG domain, G-rich domain, RNA binding domain, Zn-finger domain, and RGG-rich domains are underlined. The asterisks indicate the ALS-related mutations that occur in patients. Additional file 1: Figure S2 Large magnification of the motor neuronal cells indicating the localization of the proteins expressed in VNCs from Figure 5. Fus, Caz, and FusΔ32NLS are exclusively localized in the nucleus. FusR521G shows slightly increased staining in the cytosol, but FusΔ32 and FusNES are mainly localized in the cytoplasm. Additional file 1: Figure S3 Mutating *caz *in flies causes severe defects in the eye. (A) A female *yw *fly showing adult wild-type morphology. (B) A *caz *heterozygous female fly shows the balancer marker and intact eye structure. (C) A *caz *homozygous female fly shows growth defects in the eye and exhibits a normal body. (D) A male *yw *fly showing adult wild-type morphology. (E) A *caz *mutant male fly shows growth defects in the eye and exhibits a normal body. Mutating *caz *causes severely disrupted locomotive ability (Figure 7) without obvious changes in body size, suggesting that Caz is likely involved in neurodegeneration. Additional file 1: Table S1 Characterization of Fus toxicity in *Drosophila*. Shown here is a diagram illustrating the domain structure of Fus along with truncation and point mutation constructs that are expressed in flies by the indicated Gal4 lines. The levels of toxicity are determined in the wing (by *MS1096*-Gal4), the eye (by *GMR*Gal4), the entire body (by the *act5C*-Gal4), and the neuron (by *Elav*-Gal4 and *D42*-Gal4). More "+" symbols indicates more severe phenotypes. Click here for file

## References

[B1] Boillee S Vande Velde C Cleveland DW ALS: a disease of motor neurons and their nonneuronal neighbors Neuron 2006 52 1 39 59 10.1016/j.neuron.2006.09.018 17015226

[B2] Valdmanis PN Rouleau GA Genetics of familial amyotrophic lateral sclerosis Neurology 2008 70 2 144 152 18180444 10.1212/01.wnl.0000296811.19811.db

[B3] Rosen DR Siddique T Patterson D Figlewicz DA Sapp P Hentati A Donaldson D Goto J O'Regan JP Deng HX Mutations in Cu/Zn superoxide dismutase gene are associated with familial amyotrophic lateral sclerosis Nature 1993 362 6415 59 62 10.1038/362059a0 8446170

[B4] Sreedharan J Blair IP Tripathi VB Hu X Vance C Rogelj B Ackerley S Durnall JC Williams KL Buratti E TDP-43 mutations in familial and sporadic amyotrophic lateral sclerosis Science 2008 319 5870 1668 1672 10.1126/science.1154584 18309045 PMC7116650

[B5] Sapp PC Hosler BA McKenna-Yasek D Chin W Gann A Genise H Gorenstein J Huang M Sailer W Scheffler M Identification of two novel loci for dominantly inherited familial amyotrophic lateral sclerosis Am J Hum Genet 2003 73 2 397 403 10.1086/377158 12858291 PMC1180377

[B6] Ruddy DM Parton MJ Al-Chalabi A Lewis CM Vance C Smith BN Leigh PN Powell JF Siddique T Meyjes EP Two families with familial amyotrophic lateral sclerosis are linked to a novel locus on chromosome 16q Am J Hum Genet 2003 73 2 390 396 10.1086/377157 12840784 PMC1180376

[B7] Kwiatkowski TJ Bosco DA LeClerc AL Tamrazian E Vanderburg CR Russ C Davis A Gilchrist J Kasarskis EJ Munsat T Mutations in the FUS/TLS gene on chromosome 16 cause familial amyotrophic lateral sclerosis Science 2009 323 5918 1205 1208 10.1126/science.1166066 19251627

[B8] Vance C Rogelj B Hortobagyi T De Vos KJ Nishimura AL Sreedharan J Hu X Smith B Ruddy D Wright P Mutations in FUS, an RNA processing protein, cause familial amyotrophic lateral sclerosis type 6 Science 2009 323 5918 1208 1211 10.1126/science.1165942 19251628 PMC4516382

[B9] Belzil VV Valdmanis PN Dion PA Daoud H Kabashi E Noreau A Gauthier J Hince P Desjarlais A Bouchard JP Mutations in FUS cause FALS and SALS in French and French Canadian populations Neurology 2009 73 15 1176 1179 10.1212/WNL.0b013e3181bbfeef 19741216 PMC3462471

[B10] Corrado L Del Bo R Castellotti B Ratti A Cereda C Penco S Soraru G Carlomagno Y Ghezzi S Pensato V Mutations of FUS gene in sporadic amyotrophic lateral sclerosis J Med Genet 2010 47 3 190 194 10.1136/jmg.2009.071027 19861302

[B11] DeJesus-Hernandez M Kocerha J Finch N Crook R Baker M Desaro P Johnston A Rutherford N Wojtas A Kennelly K De novo truncating FUS gene mutation as a cause of sporadic amyotrophic lateral sclerosis Hum Mutat 2010 31 5 E1377 E1389 10.1002/humu.21241 20232451 PMC2922682

[B12] Bertolotti A Lutz Y Heard DJ Chambon P Tora L hTAF(II)68, a novel RNA/ssDNA-binding protein with homology to the pro-oncoproteins TLS/FUS and EWS is associated with both TFIID and RNA polymerase II EMBO J 1996 15 18 5022 5031 8890175 PMC452240

[B13] Zinszner H Sok J Immanuel D Yin Y Ron D TLS (FUS) binds RNA in vivo and engages in nucleo-cytoplasmic shuttling J Cell Sci 1997 110 Pt 15 1741 1750 9264461 10.1242/jcs.110.15.1741

[B14] Lagier-Tourenne C Polymenidou M Cleveland DW TDP-43 and FUS/TLS: emerging roles in RNA processing and neurodegeneration Hum Mol Genet 2010 19 R1 R46 R64 10.1093/hmg/ddq137 20400460 PMC3167692

[B15] Fujii R Okabe S Urushido T Inoue K Yoshimura A Tachibana T Nishikawa T Hicks GG Takumi T The RNA binding protein TLS is translocated to dendritic spines by mGluR5 activation and regulates spine morphology Curr Biol 2005 15 6 587 593 10.1016/j.cub.2005.01.058 15797031

[B16] Fujii R Takumi T TLS facilitates transport of mRNA encoding an actin-stabilizing protein to dendritic spines J Cell Sci 2005 118 Pt 24 5755 5765 16317045 10.1242/jcs.02692

[B17] Andersson MK Stahlberg A Arvidsson Y Olofsson A Semb H Stenman G Nilsson O Aman P The multifunctional FUS, EWS and TAF15 proto-oncoproteins show cell type-specific expression patterns and involvement in cell spreading and stress response BMC Cell Biol 2008 9 37 10.1186/1471-2121-9-37 18620564 PMC2478660

[B18] Aman P Panagopoulos I Lassen C Fioretos T Mencinger M Toresson H Hoglund M Forster A Rabbitts TH Ron D Expression patterns of the human sarcoma-associated genes FUS and EWS and the genomic structure of FUS Genomics 1996 37 1 1 8 10.1006/geno.1996.0513 8921363

[B19] Gal J Zhang J Kwinter DM Zhai J Jia H Jia J Zhu H Nuclear localization sequence of FUS and induction of stress granules by ALS mutants Neurobiol Aging 2011 32 12 2323 e27-40 20674093 10.1016/j.neurobiolaging.2010.06.010 PMC2997923

[B20] Dormann D Rodde R Edbauer D Bentmann E Fischer I Hruscha A Than ME Mackenzie IR Capell A Schmid B ALS-associated fused in sarcoma (FUS) mutations disrupt Transportin-mediated nuclear import EMBO J 2010 29 16 2841 2857 10.1038/emboj.2010.143 20606625 PMC2924641

[B21] Bosco DA Lemay N Ko HK Zhou H Burke C Kwiatkowski TJ Jr Sapp P McKenna-Yasek D Brown RH Jr Hayward LJ Mutant FUS proteins that cause amyotrophic lateral sclerosis incorporate into stress granules Hum Mol Genet 2010 19 21 4160 4175 10.1093/hmg/ddq335 20699327 PMC2981014

[B22] Feiguin F Godena VK Romano G D'Ambrogio A Klima R Baralle FE Depletion of TDP-43 affects Drosophila motoneurons terminal synapsis and locomotive behavior FEBS Lett 2009 583 10 1586 1592 10.1016/j.febslet.2009.04.019 19379745

[B23] Li Y Ray P Rao EJ Shi C Guo W Chen X Woodruff EA Fushimi K Wu JY A Drosophila model for TDP-43 proteinopathy Proc Nat Acad Sci USA 2010 107 7 3169 3174 10.1073/pnas.0913602107 20133767 PMC2840283

[B24] Ritson GP Custer SK Freibaum BD Guinto JB Geffel D Moore J Tang W Winton MJ Neumann M Trojanowski JQ TDP-43 mediates degeneration in a novel Drosophila model of disease caused by mutations in VCP/p97 J Neurosci 2010 30 22 7729 7739 10.1523/JNEUROSCI.5894-09.2010 20519548 PMC2890254

[B25] Lanson NA Jr Maltare A King H Smith R Kim JH Taylor JP Lloyd TE Pandey UB A Drosophila model of FUS-related neurodegeneration reveals genetic interaction between FUS and TDP-43 Hum Mol Genet 2011 20 13 2510 2523 10.1093/hmg/ddr150 21487023 PMC4288133

[B26] Chen Y Yang M Deng J Chen X Ye Y Zhu L Liu J Ye H Shen Y Li Y Expression of human FUS protein in Drosophila leads to progressive neurodegeneration Protein Cell 2011 2 6 477 486 10.1007/s13238-011-1065-7 21748598 PMC3563268

[B27] Wang JW Brent JR Tomlinson A Shneider NA McCabe BD The ALS-associated proteins FUS and TDP-43 function together to affect Drosophila locomotion and life span J Clin Invest 2011 121 10 4118 26 10.1172/JCI57883 21881207 PMC3195475

[B28] Immanuel D Zinszner H Ron D Association of SARFH (sarcoma-associated RNA-binding fly homolog) with regions of chromatin transcribed by RNA polymerase II Mol Cell Biol 1995 15 8 4562 4571 7623847 10.1128/mcb.15.8.4562 PMC230696

[B29] Stolow DT Haynes SR Cabeza, a Drosophila gene encoding a novel RNA binding protein, shares homology with EWS and TLS, two genes involved in human sarcoma formation Nucleic Acids Res 1995 23 5 835 843 10.1093/nar/23.5.835 7708500 PMC306767

[B30] Bischof J Maeda RK Hediger M Karch F Basler K An optimized transgenesis system for Drosophila using germ-line-specific phiC31 integrases Proc Nat Acad Sci USA 2007 104 9 3312 3317 10.1073/pnas.0611511104 17360644 PMC1805588

[B31] Jia H Liu Y Xia R Tong C Yue T Jiang J Jia J Casein kinase 2 promotes Hedgehog signaling by regulating both smoothened and Cubitus interruptus J Biol Chem 2010 285 48 37218 37226 10.1074/jbc.M110.174565 20876583 PMC2988328

[B32] Bilen J Bonini NM Drosophila as a model for human neurodegenerative disease Annu Rev Genet 2005 39 153 171 10.1146/annurev.genet.39.110304.095804 16285856

[B33] Johnson BS McCaffery JM Lindquist S Gitler AD A yeast TDP-43 proteinopathy model: Exploring the molecular determinants of TDP-43 aggregation and cellular toxicity Proc Nat Acad Sci USA 2008 105 17 6439 6444 10.1073/pnas.0802082105 18434538 PMC2359814

[B34] Hanson KA Kim SH Wassarman DA Tibbetts RS Ubiquilin modifies TDP-43 toxicity in a Drosophila model of amyotrophic lateral sclerosis (ALS) J Biol Chem 2010 285 15 11068 11072 10.1074/jbc.C109.078527 20154090 PMC2856981

[B35] Elden AC Kim HJ Hart MP Chen-Plotkin AS Johnson BS Fang X Armakola M Geser F Greene R Lu MM Ataxin-2 intermediate-length polyglutamine expansions are associated with increased risk for ALS Nature 2010 466 7310 1069 1075 10.1038/nature09320 20740007 PMC2965417

[B36] Ash PE Zhang YJ Roberts CM Saldi T Hutter H Buratti E Petrucelli L Link CD Neurotoxic effects of TDP-43 overexpression in C. elegans Hum Mol Genet 2010 19 16 3206 3218 10.1093/hmg/ddq230 20530643 PMC2908471

[B37] Wegorzewska I Bell S Cairns NJ Miller TM Baloh RH TDP-43 mutant transgenic mice develop features of ALS and frontotemporal lobar degeneration Proc Natl Acad Sci 2009 106 44 18809 18814 10.1073/pnas.0908767106 19833869 PMC2762420

[B38] Igaz LM Kwong LK Lee EB Chen-Plotkin A Swanson E Unger T Malunda J Xu Y Winton MJ Trojanowski JQ Lee VMY Dysregulation of the ALS-associated gene TDP-43 leads to neuronal death and degeneration in mice J Clin Invest 2011 121 2 726 738 10.1172/JCI44867 21206091 PMC3026736

[B39] Sun Z Diaz Z Fang X Hart MP Chesi A Shorter J Gitler AD Molecular Determinants and Genetic Modifiers of Aggregation and Toxicity for the ALS Disease Protein FUS/TLS PLoS Biol 2011 9 4 e1000614 10.1371/journal.pbio.1000614 21541367 PMC3082519

[B40] Ju S Tardiff DF Han H Divya K Zhong Q Maquat LE Bosco DA Hayward LJ Brown RH Jr Lindquist S A Yeast Model of FUS/TLS-Dependent Cytotoxicity PLoS Biol 2011 9 4 e1001052 10.1371/journal.pbio.1001052 21541368 PMC3082520

[B41] Kanning KC Kaplan A Henderson CE Motor neuron diversity in development and disease Annu Rev Neurosci 2010 33 409 440 10.1146/annurev.neuro.051508.135722 20367447

[B42] Guo W Chen Y Zhou X Kar A Ray P Chen X Rao EJ Yang M Ye H Zhu L An ALS-associated mutation affecting TDP-43 enhances protein aggregation, fibril formation and neurotoxicity Nat Struct Mol Biol 2011 18 7 822 830 10.1038/nsmb.2053 21666678 PMC3357956

[B43] Fushimi K Long C Jayaram N Chen X Li L Wu JY Expression of human FUS/TLS in yeast leads to protein aggregation and cytotoxicity, recapitulating key features of FUS proteinopathy Protein Cell 2011 2 2 141 149 10.1007/s13238-011-1014-5 21327870 PMC3093303

[B44] Kryndushkin D Wickner RB Shewmaker F FUS/TLS forms cytoplasmic aggregates, inhibits cell growth and interacts with TDP-43 in a yeast model of amyotrophic lateral sclerosis Protein Cell 2011 2 3 223 236 10.1007/s13238-011-1525-0 21452073 PMC4875312

[B45] Guttler T Madl T Neumann P Deichsel D Corsini L Monecke T Ficner R Sattler M Gorlich D NES consensus redefined by structures of PKI-type and Rev-type nuclear export signals bound to CRM1 Nat Struct Mol Biol 2010 17 11 1367 1376 10.1038/nsmb.1931 20972448

[B46] Lee BJ Cansizoglu AE Suel KE Louis TH Zhang Z Chook YM Rules for nuclear localization sequence recognition by karyopherin beta 2 Cell 2006 126 3 543 558 10.1016/j.cell.2006.05.049 16901787 PMC3442361

