# Supplementary material for: Motor neuron apoptosis and neuromuscular junction perturbation are prominent features in a Drosophila model of Fus-mediated ALS
Source: Mol Neurodegener. 2012 Mar 24;7:10. doi: 10.1186/1750-1326-7-10 (PMC3325858; doi:10.1186/1750-1326-7-10)
Supplement: Additional file 1 — Figure S1 The caz gene encodes the Drosophila homolog of Fus. A sequence alignment between Caz, mouse Fus, and human Fus is shown here. The SYQG domain, G-rich domain, RNA binding domain, Zn-finger domain, and RGG-rich domains are underlined. The asterisks indicate the ALS-related mutations that occur in patients. Additional file 1: Figure S2 Large magnification of the motor neuronal cells indicating the localization of the proteins expressed in VNCs from Figure 5. Fus, Caz, and FusΔ32NLS are exclusively localized in the nucleus. FusR521G shows slightly increased staining in the cytosol, but FusΔ32 and FusNES are mainly localized in the cytoplasm. Additional file 1: Figure S3 Mutating caz in flies causes severe defects in the eye. (A) A female yw fly showing adult wild-type morphology. (B) A caz heterozygous female fly shows the balancer marker and intact eye structure. (C) A caz homozygous female fly shows growth defects in the eye and exhibits a normal body. (D) A male yw fly showing adult wild-type morphology. (E) A caz mutant male fly shows growth defects in the eye and exhibits a normal body. Mutating caz causes severely disrupted locomotive ability (Figure 7) without obvious changes in body size, suggesting that Caz is likely involved in neurodegeneration. Additional file 1: Table S1 Characterization of Fus toxicity in Drosophila. Shown here is a diagram illustrating the domain structure of Fus along with truncation and point mutation constructs that are expressed in flies by the indicated Gal4 lines. The levels of toxicity are determined in the wing (by MS1096-Gal4), the eye (by GMRGal4), the entire body (by the act5C-Gal4), and the neuron (by Elav-Gal4 and D42-Gal4). More "+" symbols indicates more severe phenotypes. [file 1750-1326-7-10-S1.PDF]

Motor neuron apoptosis and neuromuscular junction perturbation are prominent features in a *Drosophila* model of Fus-mediated ALS

Ruohan Xia[1], Yajuan Liu[1], Liuqing Yang[2], Jozsef Gal[2], Haining Zhu[2*], and Jianhang Jia[1,2*]

[1]Markey Cancer Center, [2]Department of Molecular and Cellular Biochemistry, College of Medicine, University of Kentucky, Lexington, KY 40536, USA.

[*]Correspondence to J. Jia: Jianhang.jia@uky.edu, Phone: (859) 323-6236, Fax: (859) 257-6030; and H. Zhu: haining@uky.edu, Phone: (859) 323-3643, Fax: (859) 323-5505.

Supplemental Figure S1. The *caz* gene encodes the *Drosophila* homolog of Fus. A sequence alignment between Caz, mouse Fus, and human Fus is shown here. The SYQG domain, G-rich domain, RNA binding domain, Zn-finger domain, and RGG-rich domains are underlined. The asterisks indicate the ALS-related mutations that occur in patients.

Supplemental Figure S2. Large magnification of the motor neuronal cells indicating the localization of the proteins expressed in VNCs from Fig. 5. Fus, Caz, and FusΔ32NLS are exclusively localized in the nucleus. FusR521G shows slightly increased staining in the cytosol, but FusΔ32 and FusNES are mainly localized in the cytoplasm.

Supplemental Figure S3. Mutating *caz* in flies causes severe defects in the eye. (A) A female *yw* fly showing adult wild-type morphology. (B) A *caz* heterozygous female fly shows the balancer marker and intact eye structure. (C) A *caz* homozygous female fly shows growth defects in the eye and exhibits a normal body. (D) A male *yw* fly showing adult wild-type morphology. (E) A *caz* mutant male fly shows growth defects in the eye and exhibits a normal body. Mutating *caz* causes severely disrupted locomotive ability (Fig. 8) without obvious changes in body size, suggesting that Caz is likely involved in neuron development.

Supplemental Table S1. Characterization of Fus toxicity in *Drosophila*. Shown here is a diagram illustrating the domain structure of Fus along with truncation and point mutation constructs that are expressed in flies by the indicated Gal4 lines. The levels of toxicity are determined in the wing (by *MS1096*-Gal4), the eye (by *GMR*-Gal4), the entire body (by the *act5C*-Gal4), and the neuron (by *Elav*-Gal4 and *D42*-Gal4). More "+" symbols indicates more severe phenotypes.

Xia_Fig. S1

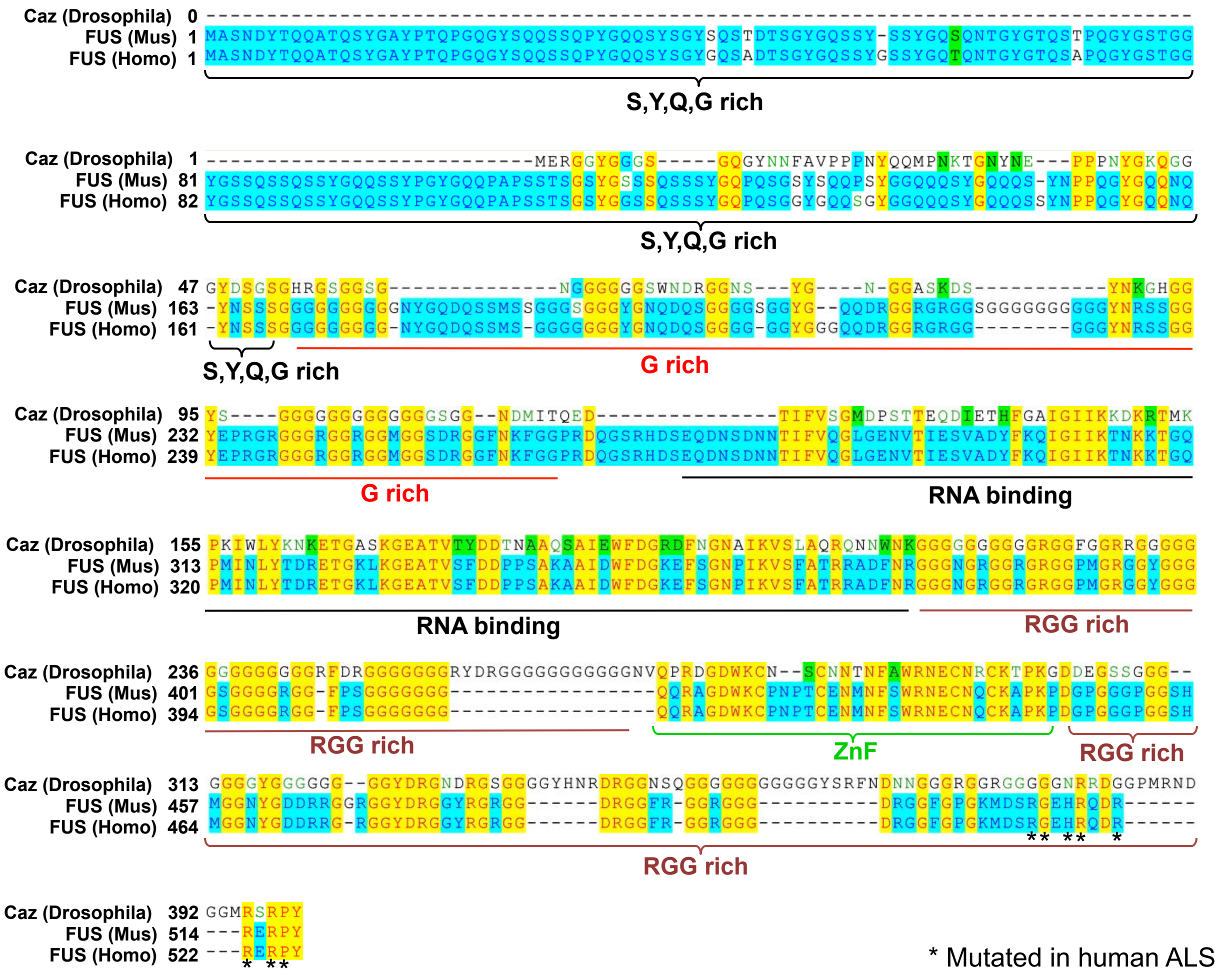

Caz (Drosophila)
FUS (Mus)
FUS (Homo)
S,Y,Q,G rich
G rich
RNA binding
RGG rich
ZnF
* Mutated in human ALS

Xia_Fig. S2

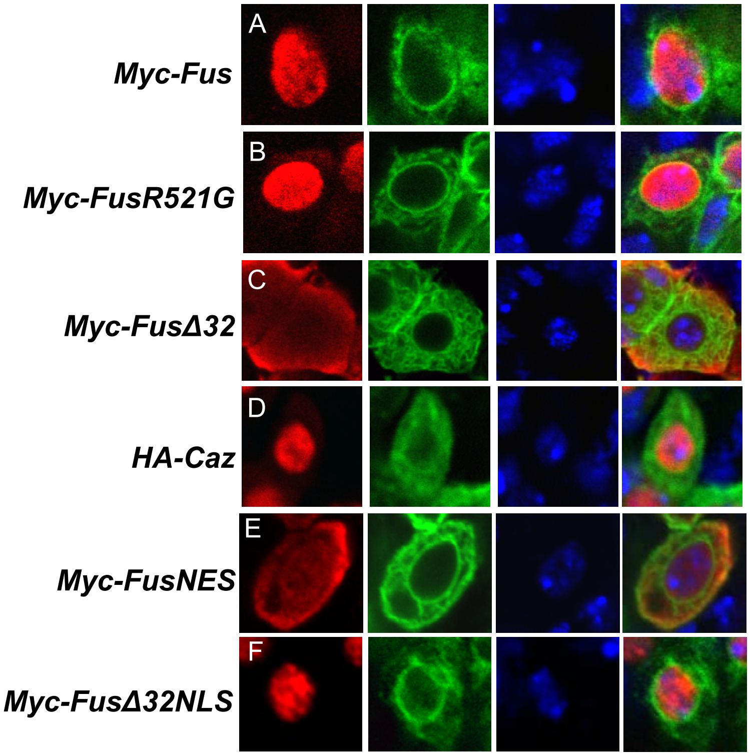
A
Myc-Fus
B
Myc-FusR521G
C
Myc-FusΔ32
D
HA-Caz
E
Myc-FusNES
F
Myc-FusΔ32NLS

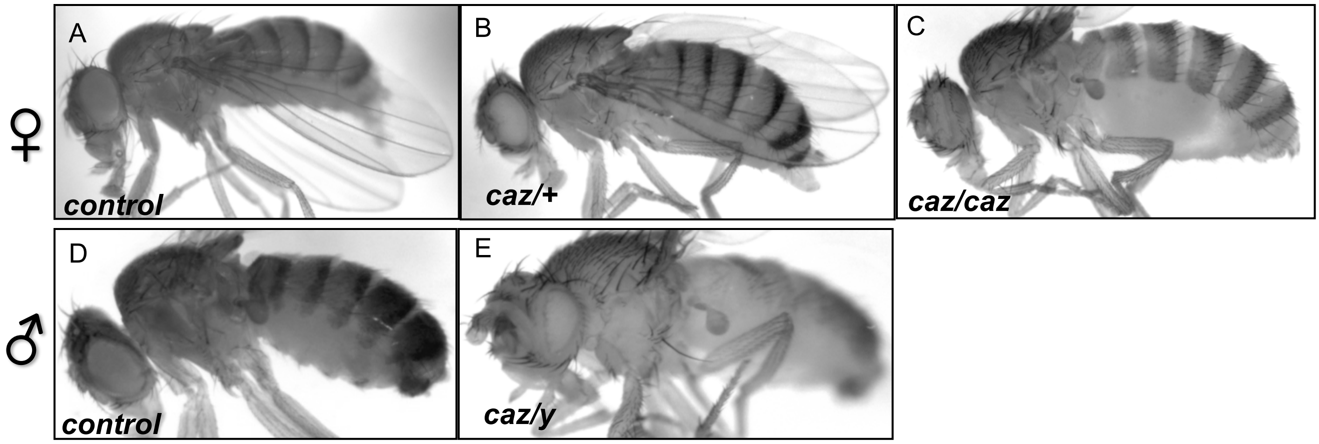
♀
♂
A
control
B
caz/+
C
caz/caz
D
control
E
caz/y

Xia_Table S1

Construct name	Diagram of the construct	Wing phenotype by *MS1096*-Gal4	eye phenotype by *GMR*-Gal4	Adult viability by *act5C*-Gal4 *Elav*-Gal4 *D42*-Gal4
**Fus**	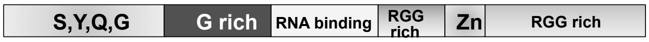	+++++	+++++	+
**FusR521G**	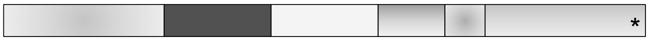	++++	++++	++
**FusR521C**	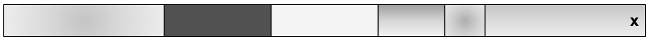	++++	++++	++
**FusΔ32**	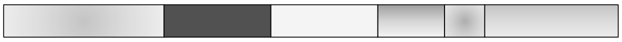	+	+	+++++
**Fus1-453**	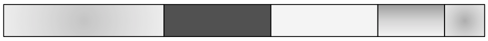	–	–	+++++
**FusY526F**	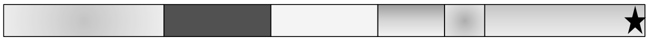	++	++	+++
**FusNES**	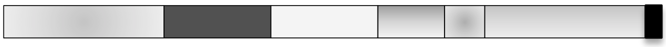	+	+	+++++
**FusΔ32NLS**	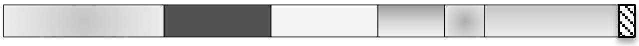	++++	++++	++
